# Absence of progression risk of chronic kidney disease in patients with urine protein-creatinine ratio below 500 mg/g: a cohort study with competing risk analysis

**DOI:** 10.3389/fmed.2025.1502597

**Published:** 2025-03-28

**Authors:** Jiang Bai, Lijuan Zhang, Jingkai Di, Wenyu Wang, Yawen Wu, Yun Zhou

**Affiliations:** ^1^The Nephrology Department of Shanxi Provincial People’s Hospital, Shanxi Medical University, Taiyuan, China; ^2^The Second Clinical Medical College, Shanxi Medical University, Taiyuan, China; ^3^School of Public Health, Shanxi Medical University, Taiyuan, China; ^4^The Fifth Clinical Medical College, Shanxi Medical University, Taiyuan, China; ^5^Department of Nephrology, Shanxi Provincial Integrated Traditional Chinese Medicine and Western Medicine Hospital, Taiyuan, China

**Keywords:** proteinuria, chronic kidney disease, progression, urine protein-to-creatinine ratio, competing risk analysis

## Abstract

**Background:**

Recent studies demonstrated a connection between minimal albuminuria and the progression of kidney disease, offering insights on the ideal threshold for antiproteinuric treatment. However, Limited data exist regarding the association between Urine Protein-Creatinine Ratio (UPCR) < 500 mg/g and Chronic Kidney Disease (CKD) progression, particularly in the context of competitive risk analysis. We aimed to investigate the correlation between UPCR and the progression of CKD in patients with UPCR below 500 mg/g.

**Methods:**

Initially, 512 patients diagnosed with stages G2-G5 of CKD and UPCR levels below 1,000 mg/g were recruited from the CKD-ROUTE cohort. Subsequently, patients with UPCR levels below 500 mg/g underwent further analysis. The Cox proportional hazards model and the competing risk model was utilized.

**Results:**

Over a median follow-up of 3.0 years, 24 out of 512 participants experienced progression of CKD. The current study revealed that compared to UPCR levels of 0–300 mg/g, patients with UPCR levels between 700 and 1,000 mg/g had a HR of 4.6 (95% CI 1.8–12.0%, *p* = 0.002), while those with UPCR levels between 300 and 700 mg/g had a HR of 2.3 (95% CI 0.9–6.2%, *p* = 0.097). Patients in the 300–500 mg/g range did not show a higher risk compared to the 0–300 mg/g category [HR = 2.5, (95% CI 0.8–8.3), *p* = 0.120]. The results of the Fine and Gray competing risk survival regression model showed the same trend.

**Conclusion:**

In patients with CKD and UPCR levels below 500 mg/g, there was no increased risk of CKD progression associated with the higher proteinuria levels.

## 1 Introduction

Proteinuria is a frequently utilized surrogate endpoint in clinical trials aimed at studying the progression of chronic kidney disease (CKD) (1). The association between increased proteinuria levels and deteriorating disease outcomes has been extensively documented in the literature (2). However, recent research has shed light on the link between even minimal levels of albuminuria (urinary albumin-to-creatinine ratios, UACR <30 mg/g) and the exacerbation of kidney disease (3, 4), prompting nephrologists to reconsider the significance of lower urinary protein levels in CKD progression. Additionally, notable meta-analyses have revealed a higher risk of kidney failure among individuals with UACR ranging from 10 to 29 mg/g compared to those below 10 mg/g (5, 6), challenging the assumption that patients with lower levels of albuminuria do not necessitate further treatment (3, 4).

Urinary protein-to-creatinine ratio (UPCR) and UACR and are employed as indicators of kidney damage and well-established prognostic factors for the development of CKD (7). In the early detection of kidney diseases, the evaluation of albuminuria proves to be more sensitive and reliable than assessing total proteinuria (8). Clinical guidelines for CKD screening and management stress the significance of utilizing albuminuria and glomerular filtration rate (GFR) for effective CKD detection (9). However, many healthcare providers still favor total urine protein evaluation due to its widespread use and cost-effectiveness compared to urine albumin measurements, which may not always be available in clinical settings (10). Moreover, urinary total protein includes specific pathological proteins, such as immunoglobulin light chains, that are pivotal in evaluating glomerular injury and the advancement of kidney disease (11). Proteinuria continues to be a valuable tool in CKD for evaluating disease severity and guiding treatment approaches (12).

To improve the comprehension of the risk gradient for CKD progression in individuals with UPCR levels below 1,000 mg/g, particularly those below 500 mg/g, this study explores the correlation between proteinuria and CKD progression in participants diagnosed with CKD within the CKD-ROUTE (The Chronic Kidney Disease Research of Outcomes in Treatment and Epidemiology) study.

## 2 Materials and methods

### 2.1 Study design and cohort participants

CKD-ROUTE study was a prospective, observational cohort study of a representative Japanese population with stage G2–G5 CKD according to the Kidney Disease Improving Global Outcomes (KDIGO) classification who were not undergoing dialysis. More than 1,000 participants were enrolled from the Tokyo Medical and Dental University Hospital and its 15 associated facilities in Tokyo, Japan. Detailed information regarding the study design has been previously published (13, 14). Participants needed to fulfill the following criteria to qualify for inclusion: (1) aged 20 years or older, (2) newly referred to or visiting the nephrology centers participating for the first time from October 2010 to December 2011, and (3) diagnosed with stage G2-G5 CKD. Individuals with a cancer diagnosis or treatment in the past 2 years, organ transplant recipients, individuals with active gastrointestinal bleeding at enrollment, and those who did not provide written informed consent were excluded from the study. All patients provided written informed consent during recruitment, and their eligibility was evaluated. This study adhered to the ethical principles outlined in the Declaration of Helsinki and received approval from the ethical committees of Tokyo Medical and Dental University, School of Medicine (No. 883), and all collaborators. Data for this study were retrieved from the “DATADRYAD” database (www.datadryad.org) in June 2023.

### 2.2 Measurements and exposures

After enrollment, an extensive data collection process commenced, involving the documentation of current prescriptions, evaluation of lifestyle behaviors (including the ability to independently feed oneself), and recording of medical histories. Furthermore, samples of blood and urine were collected for a comprehensive array of analyses, including white blood cell counts and levels of hemoglobin (Hb), albumin, urea nitrogen, creatinine, urine occult blood, urine protein, and urine creatinine (15). A modified form of the three-variable Modification of Diet in Renal Disease (MDRD) equation adjusted for the physical characteristics of Japanese individuals was utilized to estimate the glomerular filtration rate (eGFR). This adapted equation is represented as follows: eGFR = 194 × serum creatinine −1.094 × age −0.287 (if female, × 0.739) (16).

The primary exposure variable was the baseline UPCR. Initially, we performed an analysis focusing on CKD patients with UPCR levels <1,000 mg/g and subsequently those with UPCR levels <500 mg/g.

### 2.3 Outcomes

The primary endpoints of the study included CKD progression was defined as a reduction in eGFR of more than 50% or the initiation of dialysis. The clinical condition of the participants in the CKD-ROUTE trial was assessed during regular hospital visits scheduled every 6 months (17).

### 2.4 Ascertainment of covariates

Data obtained at the baseline visit included demographics, anthropometry, laboratory measurements, detailed medical history were regarded as the potential covariates, which might have confounding effects on the association between UPCR and CKD progression. All baseline laboratory measurements were conducted on samples collected during the baseline visit, employing standard assays (14, 15).

### 2.5 Statistical analysis

For skewed distributions, medians and the interquartile range (25th–75th percentile) were utilized to describe continuous variables, whereas categorical data were exhibited as relative frequencies (percentages). Missing values were imputed using k-nearest neighbors (KNN) imputation, with specific information provided in Supplementary material S1. Patients were categorized into three proteinuria groups: <300 mg/g, 300–700 mg/g, and ≥700 mg/g. Moreover, patients with a UPCR level < 500 mg/g were reclassified into two groups: 0 to <300 mg/g and 300 to <500 mg/g. Survival curves were generated, and cumulative incidences for the 2-and 3-year periods were calculated. The Cox proportional hazards model was used to estimate endpoint hazard ratios (HRs), and the Log Rank test was conducted to compare CKD progression across the entire population. In addition, the competing risk model was applied, with mortality considered as a competing risk alongside CKD progression. The subdistribution Hazard Ratio (sdHR), also known as the Fine and Gray model, was computed for CKD progression and UPCR (18). Furthermore, we examined the UPCR both as a continuous variable (log10 transformation) and categorical variables in time-to-event studies to assess the risk associated with the outcomes. The potential nonlinear relationship between UPCR and CKD progression was assessed using restricted cubic splines (RCS) through the “rms” package in R software. Knots were tested within the range of 3 to 7, and the RCS model with the lowest Akaike information criterion value was chosen.

Multivariable adjustment was conducted based on the biological and clinical plausibility of covariates as potential confounders in the relationship between UPCR and outcomes. Three primary models were constructed for covariate adjustment: Model 2, which accounted for age, sex, and body mass index; Model 3, which included the variables from Model 2 along with systolic blood pressure, hemoglobin, serum albumin, use of renin-angiotensin-aldosterone system (RAAS) inhibitor, history of cardiovascular disease, and diabetes mellitus status. Several sensitivity analyses were conducted to assess the robustness of our results. Initially, we used subdistribution hazard models to investigate the progression of CKD, treating mortality as a competing risk due to its impact on the development of CKD progression. The “cmprsk” package in R software was employed for performing the competing risk regression.

## 3 Results

### 3.1 Baseline characteristics

The baseline characteristics of the study population, categorized by UPCR levels (0 to <300, 300 to <700, and ≥700 mg/g), were detailed in Table 1. Among the 512 patients in the analyzed cohort, the average age was 71.0 years (IQR, 61.0–77.0 years), with 71.9% being men. The median UPCR was 161.0 mg/g. Participants in categories with higher UPCR levels showed elevated systolic blood pressure, lower hemoglobin, and decreased eGFR. Over a median follow-up period of 3.0 years, 24 (4.7%) individuals experienced CKD progression, and 21 (4.1%) individuals deceased. With the increasing UPCR levels, a higher rate of CKD progression was noted in the elevated UPCR groups (12.1% vs. 6.3% vs. 2.7%).

**Table 1:** Baseline characteristics of CKD-ROUTE study participants, by categories of UPCR.

	Overall	Category 1	Category 2	Category 3	*p* value
		UPCR 0 to <300 mg/g	UPCR 300 to 700 mg/g	UPCR ≥ 700 mg/g	
Participants	512	335	111	66	
Median UPCR (IQR), mg/g	165.98 [51.78, 453.13]	79.06 [32.62, 153.70]	483.38 [378.38, 566.31]	848.10 [762.38, 913.04]	<0.001
Median age (IQR), y	71.00 [61.00, 77.00]	71.00 [63.00, 77.00]	71.00 [59.00, 77.00]	71.00 [56.00, 77.00]	0.910
Male, *n* (%)	368 (71.9)	246 (73.4)	75 (67.6)	47 (71.2)	0.488
Median BMI (IQR), kg/m^2^	23.86 [21.53, 27.18]	23.72 [21.45, 26.85]	24.61 [22.21, 27.18]	23.66 [21.45, 27.13]	0.330
Median systolic blood pressure (IQR), mm/Hg	133.00 [120.00, 148.00]	130.00 [120.00, 145.00]	135.00 [123.00, 149.00]	136.00 [127.00, 150.00]	0.031
Hypertension, *n* (%)	435 (85.0)	274 (81.8)	99 (89.2)	62 (93.9)	0.015
Cardiovascular disease, *n* (%)	119 (23.2)	76 (22.7)	27 (24.3)	16 (24.2)	0.920
Diabetes, *n* (%)	142 (27.7)	91 (27.2)	31 (27.9)	20 (30.3)	0.872
RAASi, *n* (%)	301 (58.8)	195 (58.2)	63 (56.8)	43 (65.2)	0.512
Median hemoglobin (IQR), g/dL	12.70 [11.30, 14.10]	12.80 [11.70, 14.20]	12.50 [10.50, 13.80]	11.80 [10.50, 14.10]	0.005
Median albumin (IQR), g/dL	4.20 [3.90, 4.40]	4.30 [4.00, 4.40]	4.10 [3.80, 4.30]	4.10 [3.70, 4.40]	<0.001
Median eGFR (IQR), ml/min/1.73 m^2^	38.37 [25.65, 49.64]	41.35 [29.08, 50.29]	33.58 [21.58, 49.39]	29.44 [18.66, 38.37]	<0.001

Normally distributed data is presented as mean ± SD, non-normally distributed as median (IQR) and categorical variables are presented as frequencies and percentages. *p*-values for differences between groups were tested with one-way ANOVA for normally distributed data, Kruskal–Wallis test for non-normally distributed data.

CKD-ROUTE: The Chronic Kidney Disease Research of Outcomes in Treatment and Epidemiology; UPCR: urinary protein/creatinine ratio; RAAS: renin-angiotensin aldosterone system.

### 3.2 Association of UPCR and CKD progression

**Figure 1:**
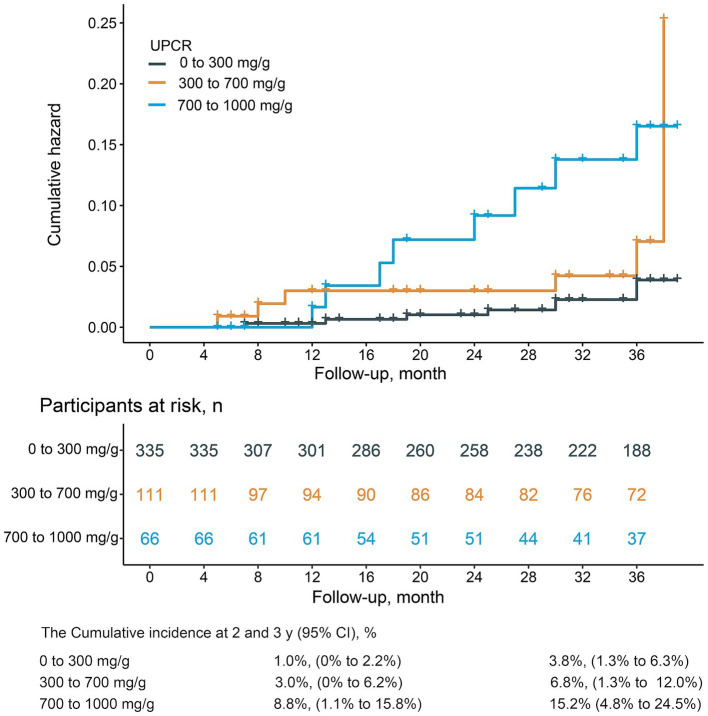
The cumulative incidences and 2- and 3-year risk differences in cumulative incidences of CKD progression among UPCR categories in CKD patients with UPCR < 1,000 mg/g.

**Figure 2:**
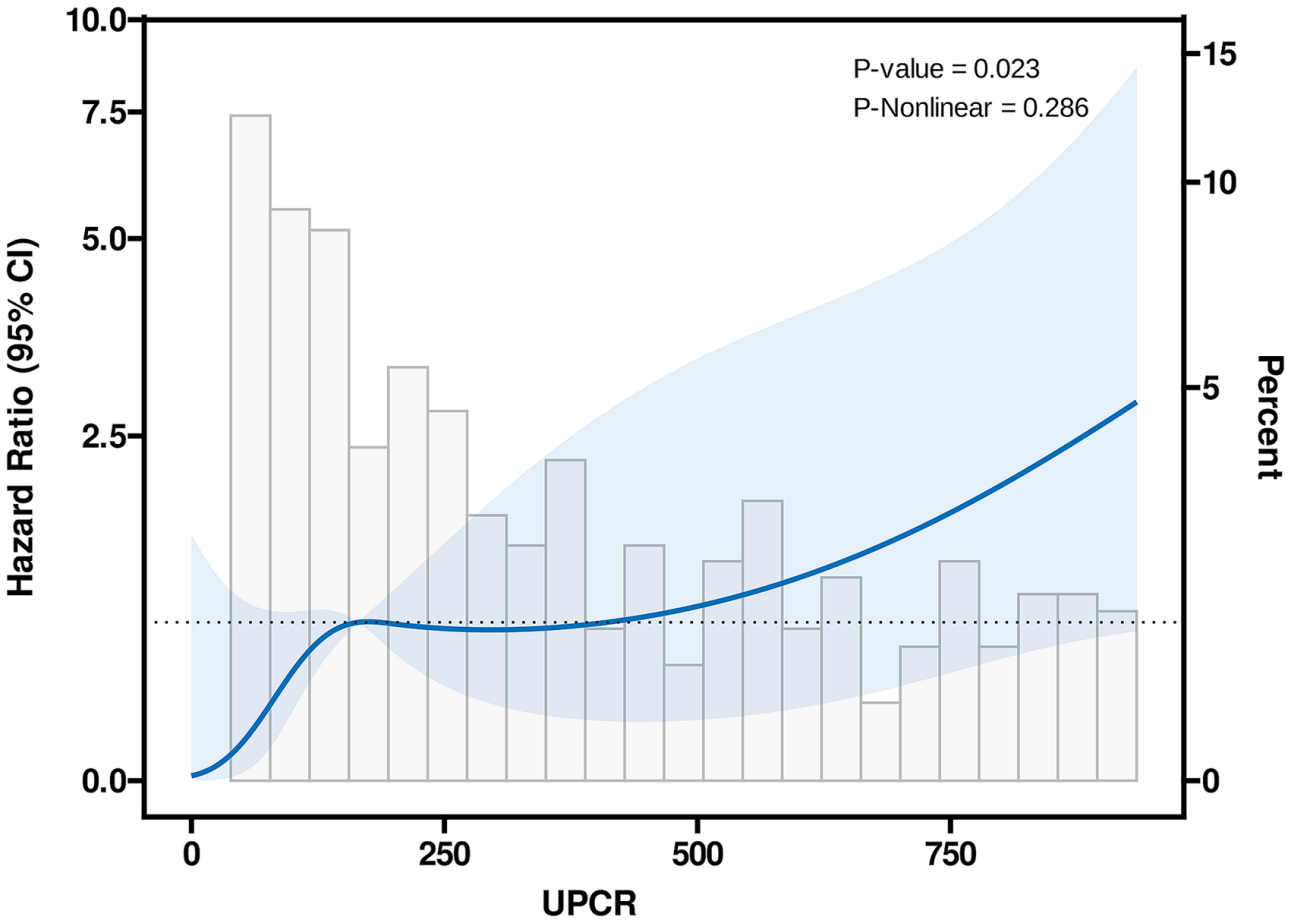
Linear association of UPCR with CKD progression in CKD patients with UPCR < 1,000 mg/g. The knots between 3 and 7 were tested respectively, and the model with lowest Akaike information criterion value was selected for RCS. Model with 4 knots located at 5th, 35th, 65th and 95th percentiles.

Figure 1 illustrated the cumulative incidences of CKD progression across categories of baseline UPCR. Over 3 years, the cumulative incidences of CKD progression were 3.8% (95% CI, 1.3–6.3%), 6.8% (95% CI, 1.3–12.0%), and 15.2% (95% CI, 4.8–24.5%) for individuals in these UPCR groups. The univariate Cox regression analysis revealed that patients with urinary UPCR levels between 300 to <700 mg/g and ≥700 mg/g exhibited HRs of 2.3 (95% CI, 0.9–6.2), *p* = 0.097 and 4.6 (95% CI, 1.8–12.0), *p* = 0.002, respectively, in comparison to those with UPCR levels of 0 to <300 mg/g (Table 2). After adjusting for covariates, the multivariable Cox regression analysis demonstrated HRs of 1.6 (95% CI, 0.6–4.4), *p* = 0.376, and 2.9 (95% CI, 1.0–8.3), *p* = 0.049 for individuals with UPCR levels of 300 to <700 mg/g and ≥ 700 mg/g, respectively, compared to those with UPCR levels of 0 to <300 mg/g (Table 2). Furthermore, patients with a UPCR level < 500 mg/g were analyzed by categorizing into 0 to <300 mg/g and 300 to <500 mg/g; patients with a UPCR between 300 to <500 mg/g did not exhibit a higher risk compared to those with UPCR of 0 to <300 mg/g in both univariate [HR = 2.5, (95% CI, 0.8–8.3), *p* = 0.120] and multivariable analysis [HR =1.8 (95% CI, 0.5–6.6), *p* = 0.362] (Table 3). Modeling UPCR with a restricted cubic spline revealed a linear association between UPCR and the overall progression of CKD (Figure 2).

**Table 2:** Association by Cox regression analysis of UPCR with CKD progression in CKD patients with UPCR < 1,000 mg/g.

UPCR (mg/g)	Continuous	Category 1 UPCR 0 to <300 mg/g	Category 2 UPCR 300 to 700 mg/g	Category 3 UPCR ≥700 mg/g
Events/total	24/512	9/335	7/111	8/66
Crude model 1	4.5 [1.6–12.9] *p* = 0.005	Reference	2.3 [0.9–6.2] *p* = 0.097	4.6 [1.8–12.0] *p* = 0.002
Multivariable model 2^a^	5.1 [1.7–15.0] *p* = 0.003	Reference	2.4 [0.9–6.4] *p* = 0.088	4.9 [1.9–12.7] *p* = 0.001
Multivariable model 3^b^	3.2 [1.0–9.9] *p* = 0.045	Reference	1.6 [0.6–4.4] *p* = 0.376	2.9 [1.0–8.3] *p* = 0.049

a Multivariable model 2: Stratified by clinical site and adjusted for age, sex and BMI.

b Multivariable model 3: Model 1+ systolic blood pressure, hemoglobin, serum albumin, use of renin-angiotensin-aldosterone system (RAAS) inhibitor, history of cardiovascular disease, and diabetes mellitus status.

**Table 3:** Association of UPCR (0 to <300 vs. 300 to <500 mg/g) with CKD progression in individuals with CKD and Normal-to-Mild Proteinuria.

UPCR (mg/g)	Category 1 UPCR 0 to <300 mg/g	Category 2 UPCR 300 to 500 mg/g
Events/total	9/335	4/60
Crude model 1	Reference	2.5 [0.8–8.3], *p* = 0.120
Multivariable model 2^a^	Reference	2.7 [0.8–8.7], *p* = 0.103
Multivariable model 3^b^	Reference	1.8 [0.5–6.6], *p* = 0.362

a Multivariable model 2: Stratified by clinical site and adjusted for age, sex and BMI.

b Multivariable model 3: Model 1+ systolic blood pressure, hemoglobin, serum albumin, use of renin-angiotensin-aldosterone system (RAAS) inhibitor, history of cardiovascular disease, and diabetes mellitus status.

### 3.3 Competing risk analysis of the association between UPCR and the progression of CKD

**Figure 3:**
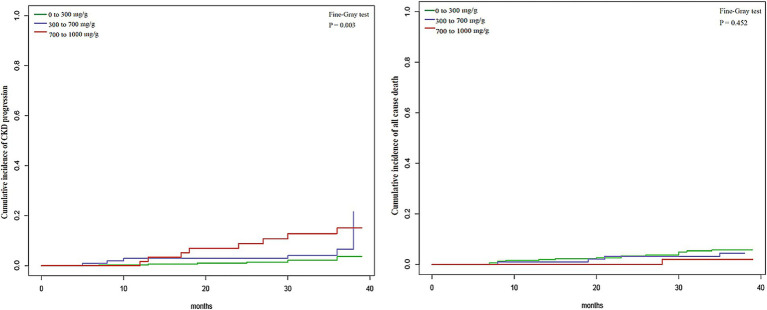
The cumulative incidence of CKD progression was estimated using the method for competing risks that accounts for the competing risk of death. The x-axes represent the months, with the y-axis on the left panel indicating the cumulative incidence of CKD progression and on the right panel indicating the cumulative incidence of all-cause death.

The Fine and Gray competing risk survival regression model was employed to investigate the association between UPCR and the progression of CKD, taking into account the competing risk of death. The results from the competing risk analysis and Cox regression analysis exhibited similar trends, indicating the reliability of the findings. The univariate competing risks analysis revealed a significant difference among the three category groups in terms of CKD progression but not for all-cause mortality (Figure 3). The results of the multivariable competing risks analysis were presented in the Supplementary Table S1. In the multivariable competing risks Model 1, patients UPCR levels of 300 to <700 mg/g and ≥700 mg/g exhibited sdHRs of 2.4 (95% CI, 0.9–6.4) and 5.1 (95% CI, 1.9–13.2) respectively, in comparison to those with UPCR levels of 0 to <300 mg/g (Supplementary Table S1). Following adjustments for covariates, patients with UPCR levels of 300 to <700 mg/g and ≥700 mg/g displayed sdHRs of 1.7 (95% CI, 0.7–4.7) and 3.2 (95% CI, 1.1–9.1) respectively, relative to those with UPCR levels of 0 to <300 mg/g in the multivariable competing risks Model 3 (Supplementary Table S1).

### 3.4 The association between UPCR and the progression of CKD across different categories

We categorized the UPCR values of 512 individuals into three equal-sized groups based on the number of participants: 0 to ≤80 mg/g, 80 to <320 mg/g, and >320 mg/g. Patients with UPCR levels of 80 to <320 mg/g and >320 mg/g exhibited HRs of 3.2 (95% CI 0.7–15.5) and 7.3 (95% CI 1.7–32.0), respectively, compared to those with UPCR levels of 0 to ≤80 mg/g (Supplementary Table S2). After adjusting for UPCR levels categorized as less than 500 mg/g and equal to or greater than 500 mg/g, or as a continuous variable, UPCR levels of 500 mg/g or higher demonstrated similar associations with the progression of CKD (Supplementary Tables S3, S4).

## 4 Discussion

The global increase in CKD incidence has been underscored in numerous studies (19–21). In 2017, the prevalence of CKD in Japan was estimated to be 13%, with crude rates of 63.1 per 1,000 individuals under 75 years old (19). The exploration of risk factors leading to kidney damage and declining kidney function has become a central focus in both the prevention and treatment of kidney diseases. A recent finding indicates that even minimal levels of albuminuria serve as risk factors for CKD progression and eventual kidney failure, challenging assumptions held by some clinicians that patients with lower albuminuria levels do not require additional treatment (3, 4). The discovery has piqued the interest of nephrologists in investigating the association between low levels of protein in urine and the progression of CKD. The present prospective cohort study found that the 3-year cumulative incidences of CKD progression were 3.8, 6.8, and 15.2% for individuals in patients with UPCR 0 to 300 mg/g, 300 to 700 mg/g and 700 to 1,000 mg/g. Among CKD patients with UPCRs <500 mg/g, there was no significant difference in CKD progression between UPCR levels of 0 to <300 mg/g and 300 to <500 mg/g. The analysis of the competing risk model across various UPCR intervals affirms the stability of the outcomes.

Proteinuria serves as both an indicator of CKD severity and a robust predictor of its progression (22). Elevated levels of proteinuria, especially exceeding 1,000 mg/g, serve as key markers signaling the progression of CKD and represent a targeted focus area for mitigating long-term kidney failure risks (23, 24). A study in France highlighted that proteinuria ≥1 g/day emerged as the most influential independent risk factor for adverse kidney outcomes (23). The majority of these studies investigated the association between proteinuria and CKD progression across the full spectrum of UPCR, particularly focusing on nephrotic-range proteinuria, utilizing either logistic regression or Cox proportional hazards regression models. Our study concentrated on individuals with CKD patients with UPCR levels <1,000 mg/g, particularly those below 500 mg/g, to explore the potential linear association between UPCR and CKD progression. Furthermore, employing competitive risk analysis, we delineated the competition between death and CKD progression to bolster result stability and clarity.

Previous studies have explored the correlation between kidney advancement in CKD patients and elevated levels of proteinuria (2). In our study, we observed that patients with UPCR levels between 700 and 1,000 mg/g exhibited a significantly higher risk (HR of 2.9) compared to those with UPCR levels between 0 and 300 mg/g. Proteinuria and the glomerular filtration rate are closely correlated with tubular atrophy, interstitial fibrosis, and scarring. Tubular epithelial cells can produce inflammatory products like reactive oxygen species and chemokines in response to the abnormal filtration of urine proteins such as complement, albumin, and cytokines (2). Subsequently, a dynamic interaction occurs between interstitial myofibroblasts and inflammatory cells in the kidney interstitium. The development of tubular atrophy, combined with nonfunctional glomeruli, results from the apoptotic loss of impaired tubular epithelia, impeding regeneration as fibrosis advances (25).

However, in the lower range of proteinuria among CKD patients, our results indicate no significant difference in the risk of progression between CKD patients with UPCR of 300 to 500 mg/g and those with UPCR of 0 to 300 mg/g. Verma et al. highlighted absolute risk variances of 7.9% (CI 3.0 to 12.7%) and 10.7% (CI 5.8 to 15.6%) among individuals with UACRs above 15 mg/g and below 5 mg/g (3). Elevated albuminuria levels within the normoalbuminuric range, as proposed by the Steno hypothesis, indicate vascular dysfunction (26). The transference of albumin to the arterial wall leads to lipid accumulation, inflammation, and the development of atherosclerosis (3, 26). Spot samples, notably measuring the UACR and UPCR, are prevalent diagnostic tools essential for accurately identifying clinically significant proteinuria crucial in the diagnosis and management of kidney diseases (27). We explored the use of UPCR to investigate the relationship between lower UPCR levels and the progression of CKD in this study. We found that among CKD patients with a UPCR below 500 mg/g, a distinct pattern emerged. When stratifying patients with a UPCR level < 500 mg/g into 0 to <300 mg/g and 300 to <500 mg/g categories, individuals in the 300 to <500 mg/g range did not demonstrate an elevated risk compared to those in the 0 to <300 mg/g group. Our study emphasized the significance of assessing the need for additional therapy in cases of lower proteinuria for clinicians. Therefore, further research is essential to confirm the relationship between lower proteinuria levels and the progression of chronic kidney disease.

Several factors may account for this discrepancy, with noteworthy differences identified between our study and others, deserving attention. In particular, within the CRIC study cohorts, individuals with low levels of albuminuria (UACR<30 mg/g) displayed significantly higher baseline eGFR levels compared to those with UPCR<1,000 mg/g in the CKD-ROUTE study (eGFR in CRIC cohorts: 49.6 mL/min/1.73 m^2^ vs. 38.4 mL/min/1.73 m^2^ in CKD-ROUTE cohorts). Additionally, the CKD-ROUTE study included a distinctive Japanese participant group, distinguishing it from other research endeavors. The impact of proteinuria on CKD progression varies among diverse ethnic populations due to various influencing factors, including genetic predispositions (28), dietary habits (29), lifestyles (29), healthcare access disparities (30), and the prevalence of comorbidities like diabetes and hypertension (31). Several studies have underscored a heightened risk of CKD advancement in African-Americans, Hispanics, and Asians compared to Caucasians (31). Furthermore, in the early detection of kidney disease, particularly at low levels of proteinuria, assessing albuminuria proves to be more sensitive and reliable compared to measuring total proteinuria (8, 10). Ultimately, a single measurement of UPCR or UACR could have impacted the results due to its day-to-day variability. Rasaratnam N discovered that UACR exhibits a considerable level of within-individual variability (32). Employing multiple urine samples for UPCR or UACR could enhance the capability to monitor changes longitudinally in clinical and research settings (32).

At lower UPCR levels, particularly those below 500 mg/g, several physiological mechanisms may contribute to the minimal progression observed in CKD. One of the primary mechanisms is the preservation of renal function due to effective renal autoregulation, which allows the kidneys to maintain stable blood flow and glomerular filtration rate (GFR) despite fluctuations in systemic conditions (33). This autoregulation may be more effective in individuals with lower UPCR levels, resulting in less renal damage over time (33). Additionally, lower UPCR levels may indicate less significant damage to the filtration barrier of the glomeruli (34). When proteinuria is minimal, there may be less injury to podocytes—cells that are essential for maintaining the integrity of the glomerular filtration barrier (34). The preservation of podocyte function is crucial for ensuring that the filtration process remains effective, thereby reducing the risk of further progression of CKD.

The strength of the study lies in its emphasis on CKD patients with UPCR <500 mg/g who did not experience CKD progression. Testing for proteinuria is straightforward and cost-effective (35). Nonetheless, many healthcare providers opt to assess total protein in urinary due to its prevalence and lower expenses, or occasionally due to challenges in analyzing urine albumin in real clinical settings. Additionally, proteinuria remains relevant in certain situations like glomerulonephritis, where it aids in evaluating the disease’s severity and determining the approach to treatment (12). We suggest that clinical practice guidelines for CKD management consider the incorporation of specific UPCR thresholds, particularly in the 300–500 mg/g range, to better stratify patient risk for disease progression. By identifying patients with lower UPCR levels who are at minimal risk, healthcare providers can tailor monitoring strategies and treatment interventions more effectively, leading to more personalized care and avoiding unnecessary overtreatment. Furthermore, our findings may serve as a foundation for enhancing existing risk assessment tools, allowing for a more nuanced understanding of CKD progression based on both proteinuria levels and other established risk factors.

This study has several limitations. UPCR was measured only at a single time point at baseline. Baseline UPCR was indicative of residual proteinuria on therapy, and it would have been higher had participants not received treatment because some of the cohort was already getting a RAASi. While we considered the use of RAASi, we did not factor in the duration of usage, potentially influencing levels of proteinuria (36). In essence, the current study is a cohort observational research, which is susceptible to residual confounding. Furthermore, to ensure reliable outcomes in multivariate Cox regression analysis, the 10-fold EPV (Events Per Variable) principle stipulates a requirement of at least 10 events (CKD progression) for each variable examined (37). Additionally, the small event rate (24 CKD progression cases among 512 participants) reduces the power of subgroup analyses, increasing the risk of false negatives or inflated hazard ratios. Interpretation of the results from multivariate regression should be approached with caution given the relatively limited number of events observed. Our attempt to focus on UPCR levels below 150 mg/g was hindered by the limited sample size and a subdued rate of CKD advancement. Additionally, the relatively small number of CKD progression events limits the statistical power of our analysis, and further validation of our findings in diverse ethnic cohorts is essential. Therefore, a comprehensive multi-center sample is essential to confirm the lower levels of proteinuria and their correlation with the progression of CKD.

In conclusion, our study demonstrated that individuals with CKD and UPCR levels <500 mg/g do not show an increased risk of CKD progression with higher levels of proteinuria. We examined the utility of UPCR to identify a consistent pattern, as exemplified by Verma et al. (3). However, our results did not show a comparable correlation between low UPCR levels and CKD progression compared to UACR. These findings underscore the necessity for additional research to ascertain if decreasing proteinuria could result in improved clinical outcomes in CKD patients, even with minimal proteinuria levels.

## Data Availability

The original contributions presented in the study are included in the article/Supplementary material, further inquiries can be directed to the corresponding author.
