# Supplementary material for: Absence of progression risk of chronic kidney disease in patients with urine protein-creatinine ratio below 500 mg/g: a cohort study with competing risk analysis
Source: Front Med (Lausanne). 2025 Mar 28;12:1502597. doi: 10.3389/fmed.2025.1502597 (PMC11985758; doi:10.3389/fmed.2025.1502597)
Supplement: Supplementary file 1 [file Data_Sheet_1.pdf]

Supplemental Material

- **Methods for imputing missing data**
- **Supplementary Table 1** Multivariable Analysis of Competing Risks Reveals Subdistribution Hazards in the Association of UPCR with CKD Progression in Patients with UPCR <1000 mg/g.
- **Supplementary Table 2** Association of UPCR (three groups of equal size) with CKD progression in individuals with CKD and UPCR <1000 mg/g.

Methods for imputing missing data

Missing values (<25% missing) were imputed based on k-nearest neighbors (kNN) [1]. The following explanatory variables were imputed: SBP (1.2% missing), BMI (11.9% missing) and albumin (0.4%missing).

Reference：

1. Liao SG, Lin Y, Kang DD, et al. Missing value imputation in high-dimensional phenomic data: imputable or not, and how?. BMC Bioinformatics. 2014;15(1):346. Published 2014 Nov 5.

- **Supplementary Table 1** Multivariable Analysis of Competing Risks Reveals Subdistribution Hazards in the Association of UPCR with CKD Progression in Patients with UPCR <1000 mg/g.

UPCR (mg/g)	Continuous	Category 1<br>UPCR 0 to <300 mg/g	Category 2<br>UPCR 300 to 700 mg/g	Category 3<br>UPCR ≥700 mg/g
Events/Total	24/512	9/335	7/111	8/66
Multivariable model 1[a]	1.002 [1.001-1.004]	Reference	2.4 [0.9-6.4]	5.1 [1.9-13.2]
Multivariable model 2[b]	1.002 [1.000-1.003]	Reference	1.7 [0.7-4.7]	3.2 [1.1-9.1]

[a]Multivariable model 1: Stratified by clinical site and adjusted for age, sex and BMI
[b]Multivariable model 2: Model 1+ use of RAASi, history of cardiovascular disease, history of diabetes, serum albumin, systolic blood pressure, hemoglobin,

Supplementary Table 2 Association of UPCR (three groups of equal size) with CKD progression in individuals with CKD and UPCR <1000 mg/g.

UPCR (mg/g)	Continuous	Category 1 UPCR 0 to <80 mg/g	Category 2 UPCR 80 to 320 mg/g	Category 3 UPCR >320 mg/g
Events/Total	24/512	2/171	7/171	15/170
Crude	1.002 [1.001-1.003]	Reference	3.2 [0.7 - 15.5]	7.3 [1.7 - 32.0]
Multivariable model 1[a]	1.002 [1.001-1.004]	Reference	3.1 [0.6 - 14.8]	7.7 [1.8 - 33.6]
Multivariable model 1[b]	1.002 [1.000-1.003]	Reference	2.7 [0.5 - 13.3]	4.8 [1.04 - 21.9]

[a]Multivariable model 1: Stratified by clinical site and adjusted for age, sex and BMI
[b]Multivariable model 2: Model 1+ use of RAASi, history of cardiovascular disease, history of diabetes, serum albumin, systolic blood pressure, hemoglobin,
