# Supplementary material for: Absence of progression risk of chronic kidney disease in patients with urine protein-creatinine ratio below 500 mg/g: a cohort study with competing risk analysis
Source: Front Med (Lausanne). 2025 Mar 28;12:1502597. doi: 10.3389/fmed.2025.1502597 (PMC11985758; doi:10.3389/fmed.2025.1502597)
Supplement: Supplementary file 2 [file Data_Sheet_2.pdf]

Supplemental Material 2

- **Supplementary Table 3** Association of UPCR (<500 vs ≥500 mg/g) with CKD progression in individuals with CKD and Normal-to-Mild Proteinuria.
- **Supplementary Table 4** Association of UPCR a continuous variable with CKD progression in individuals with CKD and Normal-to-Mild Proteinuria.

Supplementary Table 3 Association of UPCR (<500 vs ≥500 mg/g) with CKD progression in individuals with CKD and Normal-to-Mild Proteinuria.

UPCR (mg/g)	Category 1<br>UPCR 0 to <500 mg/g	Category 2<br>UPCR 500 to 1000 mg/g
Events/Total	13/395	11/117
Crude	Reference	2.7 [1.2-6.1]
Multivariable model 1[a]	Reference	3.1 [1.4-6.9]
Multivariable model 1[b]	Reference	2.0 [0.8-4.7]
Multivariable model 1[c]	Reference	1.3 [0.5-3.3]

Supplementary Table 4 Association of UPCR as a continuous variable with CKD progression in individuals with CKD and Normal-to-Mild Proteinuria.

UPCR (mg/g)	Continuous
Events/Total	24/512
Crude	1.002 [1.001-1.003]
Multivariable model 1[a]	1.002 [1.001-1.004]
Multivariable model 1[b]	1.002 [1.000-1.003]
Multivariable model 1[c]	1.001 [1.000-1.003]
